# Supplementary material for: The causal relationship between the human gut microbiota and pyogenic arthritis: a Mendelian randomization study
Source: Front Cell Infect Microbiol. 2024 Nov 26;14:1452480. doi: 10.3389/fcimb.2024.1452480 (PMC11629706; doi:10.3389/fcimb.2024.1452480)
Supplement: Supplementary file 1 [file DataSheet1.zip › mendelian test/ebi-a-GCST90027793.csv_leave_one_out.pdf]

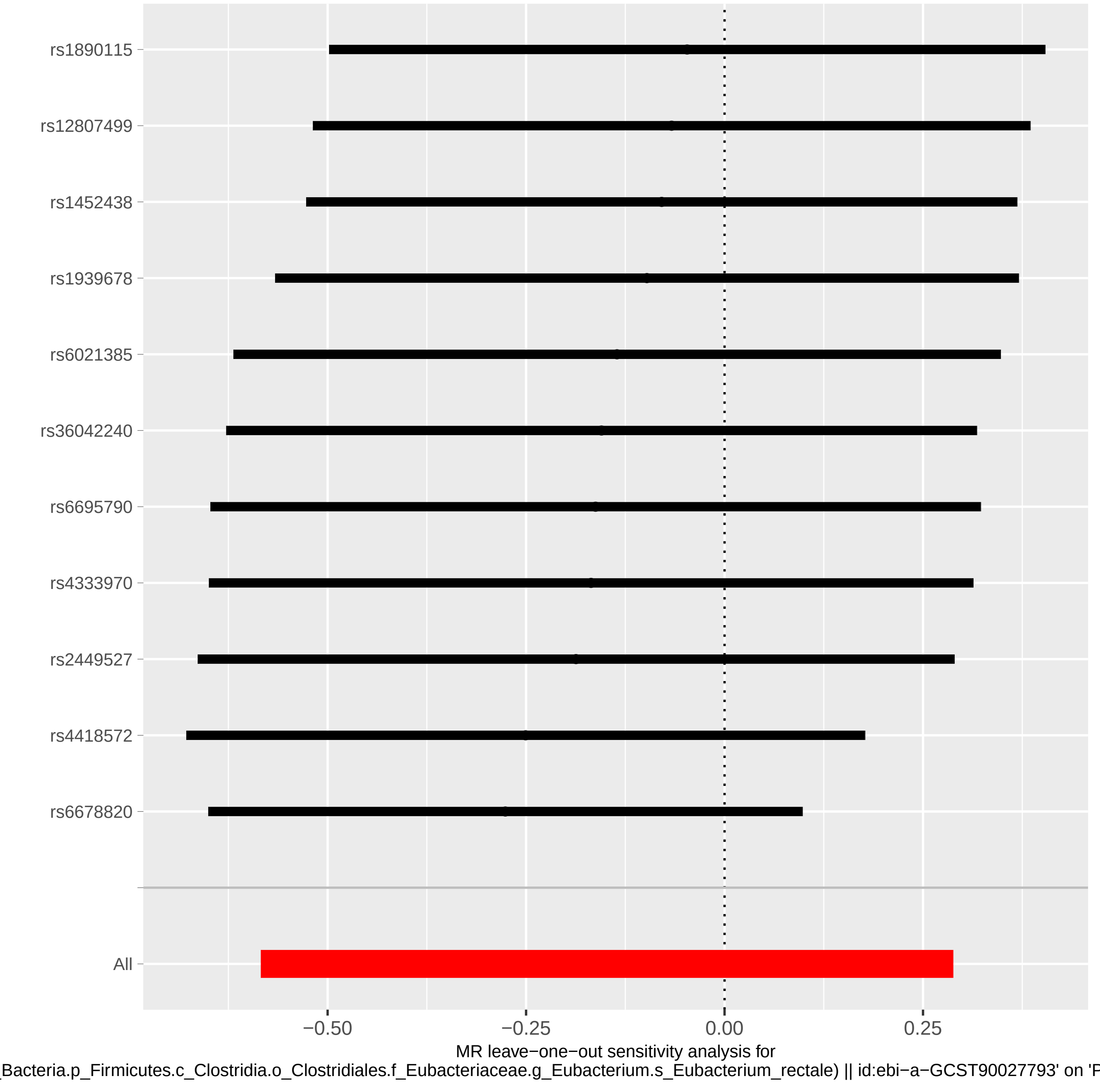
rs1890115
rs12807499
rs1452438
rs1939678
rs6021385
rs36042240
rs6695790
rs4333970
rs2449527
rs4418572
rs6678820
All
−0.50
−0.25
0.00
0.25
MR leave−one−out sensitivity analysis for
_Bacteria.p_Firmicutes.c_Clostridia.o_Clostridiales.f_Eubacteriaceae.g_Eubacterium.s_Eubacterium_rectale) || id:ebi−a−GCST90027793' on 'F
